# Elucidating the dual regulation of apoptosis and migration by Ajugasterone C in AGS cells via the PI3K/AKT pathway

**DOI:** 10.3389/fphar.2026.1823496

**Published:** 2026-07-02

**Authors:** Zejie Su, Qin Yang, Zhibin Tan, Haojie Zeng, Guoyuan Yang, Junhong Xu

**Affiliations:** 1 Department of Translational Medicine Research Center and Pharmacy, Shunde Hospital of Guangzhou University of Chinese Medicine, Foshan, Guangdong, China; 2 School of Basic Medical Sciences, Guangzhou University of Chinese Medicine, Guangzhou, Guangdong, China

**Keywords:** AGS cells, Ajugasterone C (AC), apoptosis, cell migration, molecular docking, PI3K/Akt signaling pathway

## Abstract

**Background:**

This study aimed to investigate the effects of Ajugasterone C (AC), a primary phytochemical of *Ajuga decumbens* Thunb., on human gastric cancer AGS cells and to elucidate the underlying mechanisms.

**Methods:**

The anti-proliferative effect of AC was determined using the CCK-8 assay. Its effects on clonogenic survival, apoptosis, and migration were assessed via colony formation, Annexin V-FITC/PI flow cytometry, and scratch wound healing assays, respectively. The mechanism was explored by Western blotting of key PI3K/AKT pathway proteins (PI3Kα, total AKT1, and p-AKT1). Molecular docking and dynamics simulations were employed to predict the interaction between AC and the PI3Kα protein.

**Result:**

AC treatment significantly suppressed the proliferation of AGS cells in a concentration-dependent manner, with an IC_50_ of 33.87 μM at 24 h. It markedly induced apoptosis, increasing the apoptotic rate to 30.39% ± 1.45% compared to 2.23% ± 0.13% in the control group (*P* < 0.001), and inhibited migration, reducing the scratch healing rate to 19.07% ± 8.59% versus 38.56% ± 6.85% in controls (*P* < 0.05). Furthermore, AC potently suppressed long-term clonogenicity (*P* < 0.01). Mechanistically, Western blot analysis revealed that AC dose-dependently downregulated the protein expression of PI3Kα and total AKT1, and significantly reduced AKT1 phosphorylation, leading to a substantial decrease in the p-AKT1/AKT1 activation ratio. Computational studies suggested that AC stably binds within the ATP-binding pocket of PI3Kα, providing a structural basis for its inhibitory action.

**Conclusion:**

AC exerts potent antitumor effects against gastric cancer AGS cells by inhibiting proliferation, inducing apoptosis, and suppressing migration, primarily through the inhibition of the PI3K/AKT signaling pathway. These findings suggest that AC may be a natural product with PI3K/AKT inhibitory potential, and could provide a preliminary molecular basis for further investigation into the traditional use of *Ajuga decumbensin*.

## 1 Introduction

Gastric cancer (GC), a malignancy originating from the gastric mucosal epithelium (De Nardi et al., 2026; Kotani et al., 2026), remains the fourth most prevalent cancer worldwide, exhibiting a steadily increasing incidence trend (Alsallal et al., 2026; Bautista et al., 2025). Epidemiological data from China indicate that gastrointestinal cancers contribute to nearly 45% of all cancer-related deaths (Yang et al., 2024). Recent 2025 national cancer statistics project that gastric cancer remains a severe public health threat in China, reporting an incidence of 25.41 per 100,000 and a mortality of 18.44 per 100,000, which translates to a notably low incidence-to-mortality ratio of 1.38 compared to 2.8 in the United States (Siegel et al., 2025). Currently, surgical resection combined with perioperative therapy constitutes the standard of care, offering curative potential for early-stage patients (Natti et al., 2026). However, approximately 60% of cases are diagnosed at advanced stages, where the risks of local recurrence and peritoneal metastasis are notably high (Fernández et al., 2021). Although molecular targeted therapies, such as anti-HER2 monoclonal antibodies and anti-VEGFR drugs, along with immune checkpoint inhibitors including PD-1 and PD-L1 blockers, have introduced new treatment paradigms, primary and acquired drug resistance coupled with a scarcity of effective therapeutic targets represent formidable clinical challenges that necessitate urgent investigation (Yazdi et al., 2026).

Traditional Chinese Medicine (TCM) plays a complementary role in GC treatment when integrated with Western medicine (Li Q. Y. et al., 2026; Chen et al., 2026). It enhances immunity, inhibits tumor cell invasion and proliferation, induces apoptosis and autophagy, and regulates protein expression to modulate cell proliferation and apoptosis. Clinical studies demonstrate that the Tian-Yuan-Tong-Wei decoction significantly alleviates cancer-related fatigue in patients with Qi and blood deficiency type IV GC. These findings underscore the clinical value of TCM in complementing modern oncology. Ajuga decumbens (commonly known as Jin Chuang Xiao Cao, Bai Mao Xia Ku Cao, or Jin Gu Cao) is renowned for its heat-clearing, dampness-resolving, blood-cooling, detoxifying, and pain-relieving properties. It is widely used clinically to treat sore throat, conjunctivitis, dysentery, and trauma. Studies indicate that *Ajuga decumbens* exhibits immunomodulatory, anti-fibrotic, antibacterial, anti-inflammatory, insulin-sensitizing, and antioxidant effects (Park et al., 2025; Peng et al., 2024; Teng et al., 2024; Yang et al., 2025; Zhao et al., 2025). Notably, its extracts have demonstrated antitumor activity by inhibiting hepatocellular carcinoma cells, inducing apoptosis, and promoting differentiation. The antitumor properties of *A. decumbens* are attributed to its diverse chemical constituents, including flavonoids, iridoid glycosides, and particularly, phytoecdysteroids. Among these, phytoecdysteroids, such as cyasterone and ajugasterone C, have garnered significant attention for their broad-spectrum biological activities and low toxicity (Hu et al., 2023; Karatt et al., 2023). Structurally, phytoecdysteroids share a core framework with insect molting hormones, yet they exhibit distinct pharmacological effects in mammalian systems, including potential anticancer properties. Notably, existing structure-activity relationship (SAR) studies suggest that the specific structural features of certain ecdysteroids, such as the number and position of hydroxyl groups, are critical for their cytotoxic activity against cancer cells (Singh et al., 2026; Volodin et al., 2025). For instance, analogous ecdysterones have been reported to induce cell cycle arrest and apoptosis in various cancer cell lines, including those of the liver and colon. Given that gastric cancer shares some common pathogenic features with other gastrointestinal malignancies, we hypothesize that the ecdysteroid components in Ajuga decumbens may play a pivotal role in its anti-gastric cancer effects. Ajugasterone C (AC), as a representative and highly abundant phytoecdysteroid in Ajuga decumbens, was therefore selected as the focus of this study to precisely elucidate the mechanism of action of this herb at the monomeric compound level. Inducing tumor cell apoptosis and modulating signaling pathways are recognized as key mechanisms of TCM in cancer therapy. Therefore, elucidating the specific mechanisms of TCM in GC treatment is essential to improve therapeutic efficacy, reduce recurrence rates, and prolong overall survival.

AGS cells, a gastric adenocarcinoma cell line sensitive to certain chemotherapeutic agents and radiotherapy, are widely used as a model system for studying GC and evaluating potential therapies. They are also employed in research on drug resistance mechanisms and novel therapeutic strategies related to cancer cell signaling, apoptosis, and autophagy. Studies report that inhibition of the PI3K/AKT signaling pathway induces apoptosis in AGS cells (Chen et al., 2025; Rehmutulla et al., 2025; Wan et al., 2025). The PI3K/AKT pathway, a critical oncogenic signaling axis, regulates cellular metabolism and glucose homeostasis. PI3K activation triggers AKT phosphorylation, which in turn modulates processes critical to cell survival and cell cycle progression. AKT promotes survival by inactivating pro-apoptotic factors, while its suppression inhibits GC cell proliferation and migration. Aberrant PI3K activation is closely associated with tumorigenesis.

While the antitumor potential of *A. decumbens* extracts has been recognized, the specific bioactive constituents responsible for its anti-gastric cancer activity and their precise molecular mechanisms remain poorly characterized. Furthermore, although the PI3K/AKT pathway is a well-validated therapeutic target in GC, the discovery of potent and specific inhibitors derived from natural products, particularly from traditional medicinal herbs, is still limited. Therefore, this study was designed to systematically investigate the antitumor effects of AC on human gastric cancer AGS cells and to determine whether its mechanism involves the inhibition of the PI3K/AKT signaling pathway. Our work aims to provide the first evidence characterizing AC as a novel natural product-based PI3K/AKT inhibitor, thereby bridging the traditional use of *A. decumbens* with modern molecular oncology and offering a promising lead compound for targeted therapy development.

## 2 Methods

### 2.1 Cell culture

AGS gastric cancer cells were obtained from the Cell Bank of the Representative Culture Preservation Committee of the Chinese Academy of Sciences and cultured in a constant-temperature incubator maintained at 37 °C with 5% CO_2_. Cells were grown in complete medium composed of 89% RPMI-1640, 10% fetal bovine serum (FBS), and 1% penicillin-streptomycin. The medium was replaced daily or every 2 days.

### 2.2 CCK-8 assay for half-maximal inhibitory concentration (IC_50_)

AGS cells in the logarithmic growth phase were seeded into 96-well plates at a density of 5 × 10^3^ cells/well. After 24 h, the medium was replaced with fresh complete medium containing AC at concentrations of 200 μM, 100 μM, 50 μM, 25 μM, 12.5 µM, 6.25 µM, 3.125 µM, 1.5625 µM, and 0 µM (control group), with five replicate wells per concentration. A blank group (medium only, no cells or drug) was included. After 24 h of treatment, the medium was discarded, and 100 µL of serum-free RPMI-1640 medium containing 10% CCK-8 reagent was added to each well. A 24-h treatment period was selected for initial mechanistic studies based on the IC_50_ determination and to capture early signaling and phenotypic responses. Absorbance at 450 nm was measured using a microplate reader at 1 h post-treatment. Cell viability was calculated as:

$$Cell\;viability\;\left(\%\right)=\frac{\left({OD}_{Experimental\;group}-{OD}_{blank\;group}\right)}{\left({OD}_{Control\;group}-{OD}_{blank\;group}\right)}\times 100\%$$

IC_50_ values were determined using GraphPad Prism v9.5.0.

### 2.3 Colony formation assay

AGS cells in the logarithmic growth phase were seeded into 12-well plates at 1 × 10^3^ cells/well. After 24 h, cells were treated with 35 µM AC (IC_50_ concentration) or control medium. Fresh medium was replenished every 2–3 days. Visible colonies were fixed with 1 mL of anhydrous methanol at −20 °C for 20 min, washed twice with PBS, and stained with 1 mL of 0.5% crystal violet for 30 min. Excess dye was removed by washing with distilled water. Plates were air-dried, photographed, and colonies were counted using ImageJ.

### 2.4 Scratch wound healing assay

AGS cells (3 × 10^5^ cells/well) were seeded into 6-well plates and grown to confluence. A cross-shaped scratch was created using a 200 µL pipette tip. Detached cells were removed by washing with PBS. Cells were treated with AC or control medium. Scratch areas were photographed at 0 h and 24 h using a fixed position. Relative migration rate was calculated as:

$$Migration\;rate\left(\%\right)=\frac{\left({Scratch\;area}_{0h}-{Scratch\;area}_{24h}\right)}{{Scratch\;area}_{0h}}\times 100\%$$

ImageJ was used for scratch area analysis.

### 2.5 Transwell migration assay

Cells (2 × 10^4^ cells/well) were seeded into the upper chamber of a Transwell insert (8.0 μm pore size, Corning) in 200 μL of serum-free medium. The lower chamber was filled with 600 μL of medium containing 10% FBS as a chemoattractant. After incubation at 37 °C for 24 h, non-migrated cells on the upper membrane surface were removed with a cotton swab. The migrated cells on the lower surface were fixed with 4% paraformaldehyde for 15 min and stained with 0.1% crystal violet for 10 min. Five random fields per well were photographed under an inverted microscope, and the number of migrated cells was quantified using ImageJ software.

### 2.6 Apoptosis assay

AGS cells (3 × 10^5^ cells/well) were seeded into 6-well plates and treated with AC for 24 h when cell density reached 60%–70% confluence. Floating and adherent cells (harvested using trypsin) were pooled, stained with Annexin V-FITC/PI (Apoptosis Detection Kit), and analyzed by flow cytometry.

### 2.7 Western blotting

Cells were lysed on ice using RIPA buffer containing 1% protease inhibitor cocktail. Protein concentrations were quantified via BCA assay. Proteins were separated by SDS-PAGE, transferred to PVDF membranes, and probed with primary antibodies (PI3Kα, AKT1, p-AKT1 [Ser473]; 1:1,000 dilution) at 4 °C overnight. Membranes were washed with TBST, incubated with secondary antibodies (1:5,000) for 1 h at room temperature, and visualized using ECL. Band intensities were quantified using ImageJ.

### 2.8 Molecular docking

The 3D structure of AC (PubChem: MOL007751) was converted to PDB format using Open Babel GUI 3.1.1. The crystal structure of PI3Kα (PDB ID: 8EXL) was preprocessed in PyMOL 2.5.2 by removing ligands, water molecules, and adding hydrogen atoms. Active site coordinates were defined in AutoDock Tools 1.5.7 (El-Hachem et al., 2017). Semi-flexible docking was performed with a grid spacing of 0.375 Å. The optimal binding conformation (lowest binding free energy) was visualized in PyMOL to analyze hydrogen bonds and hydrophobic interactions.

### 2.9 Molecular dynamics (MD) simulations

The AC-PI3Kα complex was solvated in a TIP3P water box and neutralized with Na^+^/Cl^−^ ions. All MD simulations were performed using the AMBER20 package (Lee et al., 2020; Zhang et al., 2021). The system underwent a two-step minimization protocol: initially restraining the solute, followed by full-system minimization. Subsequently, the system was gradually heated from 0 K to 310 K (37 °C) under the NVT ensemble, followed by a 5 ns equilibration under the NPT ensemble to ensure proper density and pressure. A 100 ns production MD simulation was then conducted. The stability of the simulation was assessed by calculating the root-mean-square deviation (RMSD) of the protein Cα-atoms and the radius of gyration (Rg). Furthermore, the per-residue flexibility was evaluated by calculating the root-mean-square fluctuation (RMSF) of the protein backbone atoms throughout the simulation trajectory, allowing for the identification of flexible loops versus stable secondary structures.

### 2.10 Statistical analysis

Data are expressed as mean ± SD (x ± s). Statistical significance was determined using Student’s t-test in GraphPad Prism v9.5.0. Significance levels were defined as *P < 0.05, **P < 0.01 and ***P < 0.001.

## 3 Result

### 3.1 CCK8 assay reveals concentration-dependent growth inhibition

**FIGURE 1:**
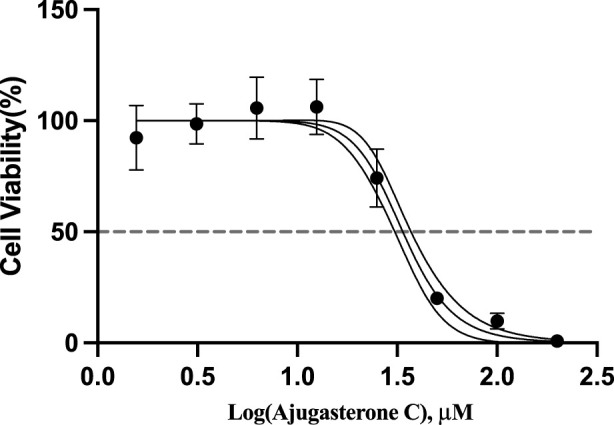
Inhibitory effect of AC on the proliferation of AGS cells. The X-axis represents the logarithm of the Ajugasterone C concentration (Log[Ajugasterone C, µM]), and the Y-axis indicates the percentage of cell viability relative to the untreated control. The data points (dots) represent the mean values with 5 biological replicates per group (n = 5), and the solid curve is the fitted dose-response curve. The region between two black dashed lines indicates the 95% confidence interval of the fit. The grey dashed line marks the 50% viability level. The intersection of this dashed line with the fitted curve determines the half-maximal inhibitory concentration (IC_50_).

The inhibitory effect of AC on the proliferation of AGS gastric cancer cells was quantitatively evaluated using the CCK8 assay. As shown in Figure 1, after 24 h of drug intervention, cell viability exhibited a significant concentration-dependent decline, with a half-maximal inhibitory concentration (IC_50_) of 33.87 ± 6.15 μM at 24 h (Figure 1). Notably, prolonged exposure resulted in enhanced cytotoxicity, with the IC_50_ decreasing to 29.41 ± 2.60 μM at 48 h (Supplementary Figure S1A) and further dropping to 11.91 ± 1.12 μM at 72 h (Supplementary Figure S1B), indicating a strong time-dependent inhibitory effect. To investigate whether AC exerts similar anti-proliferative effects on other gastric cancer cell lines, we extended our evaluation to HGC-27 cells. The results demonstrated that AC also effectively inhibited the proliferation of HGC-27 cells, yielding a 24-h IC_50_ of 34.10 ± 1.26 µM (Supplementary Figure S1C). Based on the dose-response relationship and experimental feasibility, subsequent experiments uniformly adopted a standard working concentration of 34 μM, approximating the IC_50_ value.

### 3.2 Flow cytometry confirms apoptosis-inducing effect

**FIGURE 2:**
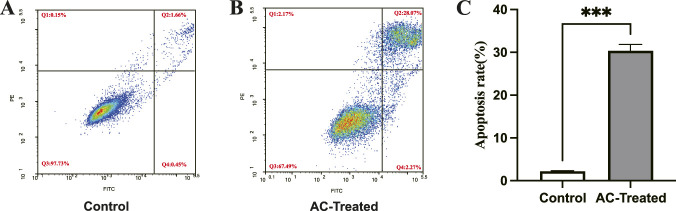
AC induces apoptosis in AGS cells detected. **(A)** Representative dot plot of the control group. The lower right quadrant (Annexin V+/PI-) represents early apoptotic cells, and the upper right quadrant (Annexin V+/PI+) represents late apoptotic cells. **(B)** Representative dot plot of AGS cells treated with 34 µM AC for 24 h. **(C)** Quantitative analysis of the total apoptosis rate (early + late apoptosis). Data are presented as mean ± SD from three independent replicates (n = 3). Statistical analysis was performed using a t-test, and *** indicates *P* < 0.001 compared to the control group.

To investigate the impact of AC on apoptosis, cells in each group were subjected to Annexin V-FITC/PI double staining for apoptosis detection. Flow cytometry analysis revealed that the apoptosis rate in the experimental group significantly increased to 30.39% ± 1.45%, compared to 2.23% ± 0.13% in the control group, showing a statistically significant difference (***P < 0.001, Figure 2). These results indicate that AC exerts its antitumor effect by inducing apoptosis.

### 3.3 Scratch assay reveals inhibition of migration capacity

**FIGURE 3:**
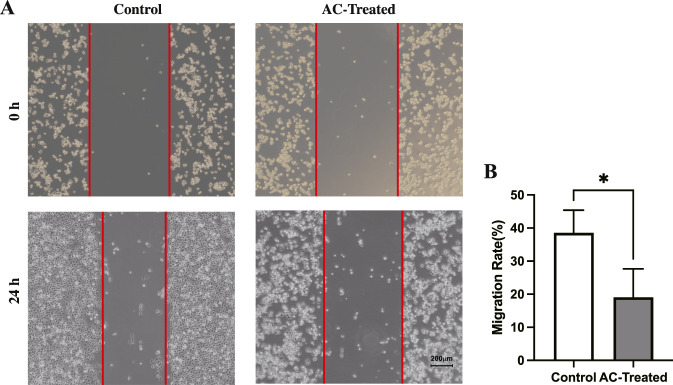
AC inhibits the migration of AGS cells. **(A)** Representative microscopic images (scale bar: 200 µm) of the scratch wound at 0 h and 24 h for the control and AC-treated (34 µM) groups. The red dashed lines indicate the wound edges. **(B)** Quantitative analysis of the relative migration rate. Data are presented as mean ± SD from three independent replicates (n = 3). Statistical analysis was performed using a t-test, and * indicates *P* < 0.05 compared to the control group.

The effect of AC on the migration of AGS gastric cancer cells was assessed using a scratch wound healing assay. As shown in Figure 3, after 24 h of treatment with 34 μM AC, the relative migration rate of the experimental group decreased to 19.07% ± 8.59%, significantly lower than that of the control group (38.56% ± 6.85%), with a marked statistical difference (*P < 0.05, Figure 3). Furthermore, the inhibitory effect on cell migration was corroborated by the Transwell migration assay. Quantitative analysis revealed that AC treatment significantly suppressed the chemotactic motility of AGS cells (***P < 0.001, Supplementary Figure S2). These convergent results from both assays demonstrate that this compound effectively inhibits the lateral migration of AGS cells, suggesting its potential interference with tumor invasion and metastasis.

### 3.4 Colony formation assay validates long-term proliferation suppression

**FIGURE 4:**
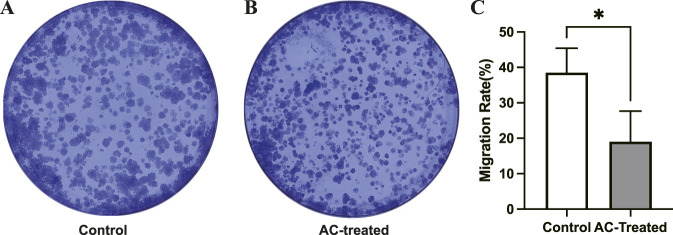
AC inhibits the long-term clonogenic survival of AGS cells. **(A)** Representative images of crystal violet-stained colonies formed by AGS cells from the control and **(B)** AC-treated (35 μM) groups. **(C)** Quantitative analysis of the number of colonies. Data are presented as mean ± SD from three independent replicates (n = 3). Statistical analysis was performed using a t-test, and * indicates *P* < 0.05 compared to the control group.

Based on the IC_50_ value, the long-term proliferative potential of cells was evaluated via a plate colony formation assay to detect the effect of AC on AGS cell clonogenicity. As shown in Figure 4, the number of colonies formed by AGS cells in the experimental group was significantly reduced compared to the control group (**P < 0.01), indicating that AC not only induces acute cytotoxicity but also persistently suppresses the self-renewal capacity of tumor cells.

### 3.5 Western blot elucidates PI3K/AKT pathway regulatory mechanism

**FIGURE 5:**
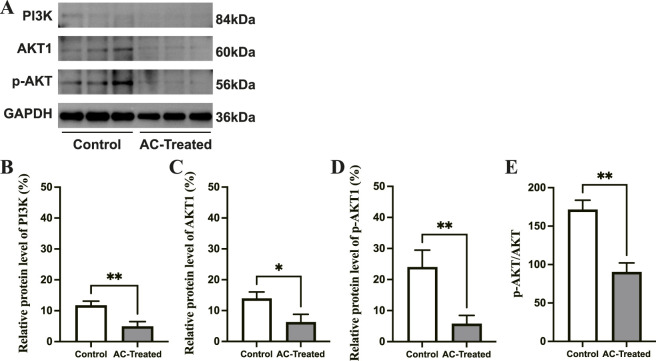
AC inhibits the PI3K/AKT signaling pathway in AGS cells. **(A)** Representative immunoblots of PI3Kα, total AKT1, phosphorylated AKT1 (p-AKT1 at Ser473), and the loading control GAPDH in AGS cells treated with increasing concentrations of AC. **(B–D)** Densitometric quantification of the protein levels of PI3Kα **(B)**, total AKT1 **(C)**, and p-AKT1 **(D)**, normalized to GAPDH. Data are expressed as a percentage of the control group and presented as mean ± SD from three independent replicates (n = 3). Statistical analysis was performed using a t-test, *P < 0.05, **P < 0.01. **(E)** The ratio of p-AKT1 to total AKT1, indicating AKT activation levels. Data are presented as mean ± SD from three independent replicates (n = 3). Statistical analysis was performed using a t-test.AC treatment significantly decreased the p-AKT1/AKT1 ratio.

Given the pivotal role of the PI3K/AKT signaling pathway in gastric cancer progression, we further investigated whether AC exerts its effects through this pathway. Western blot analysis (Figure 5; Table 1) revealed that the relative protein expression levels of PI3Kα, AKT1, and its phosphorylated form p-AKT1 in the experimental group decreased to 4.89% ± 1.59% (vs. 11.71% ± 1.40% in the control), 6.23% ± 2.67% (vs. 13.90% ± 2.15%), and 5.75% ± 2.71% (vs. 29.98% ± 5.50%), respectively. Notably, the p-AKT1/AKT1 ratio dropped significantly from 171.3% in the control group to 90.18% (*P* < 0.01), suggesting that AC may inhibit the expression of PI3Kα and AKT1 proteins, as well as AKT1 phosphorylation, thereby effectively blocking the PI3K/AKT signaling pathway.

**TABLE 1:** Protein levels and phosphorylation ratio of PI3K/AKT pathway.

Group	PI3Kα/GAPDH	p-AKT1/GAPDH	AKT1/GAPDH	(p-AKT1/GAPDH)/ (AKT1/GAPDH)
Control	11.71% ± 1.40%	29.98% ± 5.50%	13.90% ± 2.15%	171.3% ± 12.47%
Treated	4.89% ± 1.59%	5.75 ± 2.71	6.23% ± 2.67%	90.18% ± 11.89%

### 3.6 Molecular dynamics simulations reveal the stable binding of AC to PI3Kα

**FIGURE 6:**
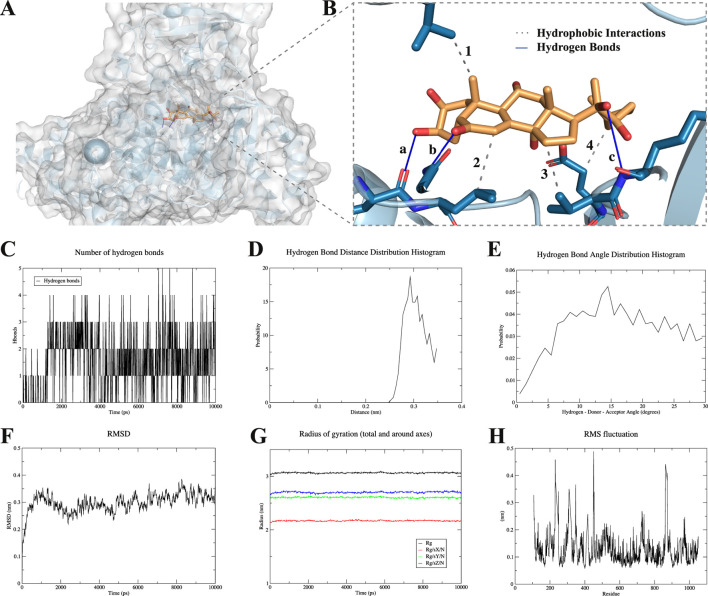
Molecular dynamics simulation analysis of the ligand-protein complex structure and stability. **(A)** Overall structure of the complex. The protein is displayed as a semi-transparent molecular surface and the bound ligand is shown as a ball-and-stick model within the binding pocket. The dashed box highlights the interaction site detailed in panel B. **(B)** Close-up view of key interactions between the ligand (light brown sticks) and protein residues (dark blue sticks). Specific hydrophobic interactions are indicated by gray dashed lines with key sites labels (1, 2, 3, 4), and hydrogen bonds are shown as blue dashed lines with key sites labeled (a, b, c). **(C)** Time evolution of the number of hydrogen bonds formed between the ligand and the protein throughout the simulation trajectory. **(D)** Distribution profile of donor-acceptor distances for the hydrogen bonds observed during the simulation. **(E)** Distribution profile of the angles (D-H···A) for the hydrogen bonds observed during the simulation. **(F)** RMSD of the protein backbone atoms relative to the initial structure, plotted as a function of simulation time, reflecting the overall structural stability. **(G)** Time evolution of the Rg of the complex along different axes, indicating changes in overall compactness and shape. **(H)** RMSF per residue for the protein backbone, highlighting regions of high flexibility (peaks) and low flexibility (troughs) during the simulation.

To complement our experimental findings and gain insights into the direct interaction between AC and PI3Kα, we performed MD simulations. Initial molecular docking predicted that AC snugly binds within the ATP-binding pocket of PI3Kα (Figure 6A), a canonical binding site shared by classical PI3K inhibitors (Supplementary Figures S3–S5; Supplementary Tables S1–S7). The binding is primarily stabilized by a network of interactions: a strong hydrogen bond, multiple hydrophobic contacts, and additional hydrogen bonds (Figure 6B). Specifically, the side chain of GLN928A forms a hydrogen bond with the ligand exhibiting optimal geometry and strong electrostatic interaction, serving as a key anchoring point (Figures 6C–E; Table 2). Additional stability is provided by hydrogen bonds involving the backbone atoms of MET833A and ARG852A (Figures 6C–E; Table 3). Furthermore, the hydrophobic side chains of LEU279A, LEU834A, VAL851A, and GLU849A collectively form a hydrophobic cavity, engaging in extensive van der Waals contacts with the ligand, which significantly enhances binding affinity (Table 3). Notably, the RMSD of the protein backbone stabilized below 2 Å after an initial 20 ns equilibration, indicating a robust and persistent interaction (Figure 6F). Analysis of the Rg for the complex further confirms its structural stability. As shown in the corresponding figure, the total Rg and its components along the three principal axes (Rg/sX/N, Rg/sY/N, Rg/sZ/N) remained remarkably stable throughout the entire 100 ns simulation. The total Rg fluctuated minimally around a mean value of approximately 2.5 nm, indicating the overall compactness and three-dimensional fold of PI3Kα were maintained upon ligand binding. The stability of the individual axial components demonstrates the complex did not undergo significant anisotropic deformation (Figure 6G). The mechanistic insight into AC’s inhibitory potential was revealed by analyzing the binding interface. Crucially, AC formed a stable hydrogen bond network with residues instrumental in catalysis, specifically MET833, ARG852, and GLN928. The distance profiles (Figure 6D) showed that the hydrogen bond with GLN928 remained stable throughout the simulation, a residue known to be vital for ATP binding in PI3Kα. Additionally, extensive van der Waals contacts with the hydrophobic pocket provided the driving force for high-affinity binding. These simulations suggest that AC functions as a structural mimic of ATP, occupying the catalytic cleft and potentially sterically hindering substrate access. Furthermore, the RMSF analysis (Figure 6H) indicates that the majority of the protein backbone exhibited low fluctuations, suggesting that the binding of AC does not induce significant local structural disruptions or increase the flexibility of the overall protein scaffold.

**TABLE 2:** Hydrogen Bonds between the ligand and receptor.

Index	Residue	Amino	Distance H-A	Distance D-A	Donor angle	Donor atom	Acceptor atom
a	833A	MET	2.54	3.26	132.15	34 [O3]	6960 [O2]
b	852A	ARG	2.95	4.07	156.15	7114 [Nam]	30 [O3]
c	928A	GLN	1.85	3.03	169.04	7794 [Nam]	28 [O2]

**TABLE 3:** Hydrophobic Interactions between the ligand and receptor.

Index	Residue	Amino	Distance	Ligand atom	Protein atom
1	279A	LEU	3.38	19	1602
2	834A	LEU	3.67	7	6971
3	849A	GLU	3.56	27	7093
4	851A	VAL	3.44	10	7111

## 4 Discussion

This study provides evidence that AC, a primary active component of *Ajuga decumbens* Thunb. (Herba Ajugae), exerts antitumor effects against human gastric cancer AGS cells, with its actions associated with the modulation of the PI3K/AKT signaling pathway. Experimental results indicate that AC suppressed cell proliferation *in vitro*, with a 24-h IC_50_ of 33.87 μM. Furthermore, AC treatment promoted apoptosis, increasing the rate from 2.23% to 30.39%, and inhibited cell migration, reducing the scratch healing rate from 38.56% to 19.07%. Western blot analysis revealed that AC downregulated the expression of both PI3Kα and total AKT1, and attenuated the phosphorylation level of AKT1 (Ser473), leading to a decrease in the p-AKT1/AKT1 activation ratio from 171.3% to 90.18%. Collectively, these results are consistent with the interpretation that AC may exert its antitumor activity through a mechanism linked to the suppression of PI3K/AKT pathway activity.

Integrating cellular assays with atomic-level molecular dynamics simulations (Sauve et al., 2026; Zhang C. et al., 2026), our data suggest a potential mechanism by which AC interacts with PI3Kα. The simulations indicate that AC can stably bind within the ATP-binding pocket of PI3Kα. The stable binding of AC within the ATP-binding pocket of PI3Kα, evidenced by low RMSD values and favorable binding free energy, provides a structural rationale for it to act as a potential direct ATP competitor. This model aligns with the observed sharp reduction in AKT phosphorylation, while the concomitant decrease in total PI3Kα protein levels likely represents a secondary effect. Based on these integrated findings, we propose a dual mode of action. Primarily, by occupying the catalytic site, AC appears to function as a competitive inhibitor, potentially impeding PI3Kα kinase activity and thus preventing AKT phosphorylation. Secondarily, the prolong and stable binding of AC may allosterically compromise protein stability, potentially triggering ubiquitination and proteasomal degradation pathways, which would account for the observed reduction in total PI3Kα protein levels observed in our Western blot analysis. Regarding the observed reduction in total PI3Kα protein levels, the MD data offers a speculative perspective: the sustained and stable binding of AC might induce subtle allosteric changes or steric clashes that expose hydrophobic patches, potentially flagging the protein for ubiquitination and subsequent proteasomal degradation. Alternatively, the disruption of the PI3K/AKT signaling loop itself could trigger feedback mechanisms affecting protein stability. Thus, the computational data supports a dual-action model: a direct competitive blockade of enzymatic activity coupled with a secondary effect on protein turnover. While the cellular assays demonstrated a decrease in both phospho-AKT and total PI3Kα levels, the MD simulations provide a plausible structural explanation for the former. The persistent occupancy of the ATP-binding pocket by AC, anchored by key residues like GLN928, suggests a competitive inhibition mechanism that directly impedes kinase activity, leading to the sharp drop in AKT phosphorylation. Regarding the observed reduction in total PI3Kα protein levels, the MD data offers a speculative perspective: the sustained and stable binding of AC might induce subtle allosteric changes or steric clashes that expose hydrophobic patches, potentially flagging the protein for ubiquitination and subsequent proteasomal degradation. Alternatively, the disruption of the PI3K/AKT signaling loop itself could trigger feedback mechanisms affecting protein stability. Thus, the computational data supports a dual-action model: a direct competitive blockade of enzymatic activity coupled with a secondary effect on protein turnover.

The PI3K/AKT pathway is a well-established oncogenic driver in gastric cancer, with its central role in tumor progression, metastasis, and immune evasion continuously underscored, make it a focal point for therapeutic development (Fattahi et al., 2020; Li H. et al., 2026; Lin et al., 2026; Wei et al., 2026; Zhang T. et al., 2026; Duan et al., 2025). A growing body of research has identified this pathway as a common target for various natural products. For instance, triterpenoids, ginsenoside CK, and flavonoids like didymin have all been reported to exert anti-gastric cancer effects by modulating PI3K/AKT signaling. However, many of these studies primarily demonstrate an association with pathway activity without delineating the precise molecular initiation point or the fine-tuned regulation of downstream signaling events. In contrast, our findings provide further mechanistic insight: we show that the natural compound AC potently impacts the PI3Kα isoform and reduces its protein levels, which is frequently implicated in solid tumors. This action correlates with a reduction in the generation of the key secondary messenger PIP3 and, consequently, precisely inhibits the downstream phosphorylation cascade of AKT, including both the PDK1-mediated phosphorylation at Thr308 and the mTOR-dependent modification at Ser473. Therefore, this work not only places AC within the class of natural products that modulate the PI3K/AKT pathway but, more importantly, delineates a clear mechanistic cascade from a specific isoform target to key phosphorylation events. This detailed elucidation deepens the understanding of the mode of action for such natural compounds and reinforces the consensus that the PI3K/AKT axis is a critical and actionable node for intervention in gastric cancer.

The observed downregulation of p-AKT1 (Ser473) by AC may correlate with impaired mTORC2 complex stability or feedback activation of the PP2A phosphatase (Wang N. et al., 2026), as reported for other AKT allosteric inhibitor MK-2206 (Dai et al., 2026; Wang J. et al., 2026). The inactivation of this pathway likely orchestrates a multifaceted anti-tumor response. Specifically, the induction of apoptosis is mechanistically driven by the relief of AKT-mediated inhibitory phosphorylation on pro-apoptotic proteins like Bim and BAD, which initiates the mitochondrial apoptotic cascade (Jiménez-Vacas et al., 2025). Concurrently, the suppression of cell migration aligns with the established role of PI3K/AKT inhibitor in downregulating key EMT transcription factors and matrix-degrading enzymes, thereby impeding metastatic dissemination (Monisha Yadav et al., 2026; Mazumdar and Biswas, 2026; Srivastava et al., 2026). This downregulation consequently reverses the EMT program by restoring epithelial marker expression and suppressing mesenchymal markers, alongside reducing the secretion of matrix-degrading enzymes such as MMP-9, thereby inhibiting extracellular matrix (ECM) degradation and impeding metastatic dissemination (Monisha Yadav et al., 2026; Mazumdar and Biswas, 2026; Bai et al., 2026). These events, combined with cell cycle arrest via FOXO3a-mediated p27 upregulation and Cyclin D1/CDK4 inhibition, illustrate the synergistic anticancer effects of PI3K/AKT pathway inactivation.

As the first study to explore the anti-gastric cancer activity of AC, several limitations inherent to this preliminary investigation must be acknowledged, which also define clear avenues for future research. First, the current findings are based solely on *in vitro* experiments using the AGS cell line. Validating in additional GC cell lines and in relevant *in vivo* models is imperative to confirm broader applicability and therapeutic potential. Second, the absence of parallel experiments using a reference PI3K/AKT inhibitor limits direct benchmarking of AC’s inhibitory potency and specificity against this pathway. Future studies incorporating such pharmacological controls are essential. Third, although our approach provides correlative evidence, establishing a definitive causal link necessitates further mechanistic investigation. Rescue experiments, such as reconstituting pathway activity through overexpression of constitutively active AKT or mutant PI3KCA in the presence of AC, would be crucial to solidify the mechanistic claim. Finally, considering that AC is a component of a complex botanical extract, the potential synergistic effects with other constituents present in *A. decumbens* Thunb. and their multi-target interaction networks warrant in-depth exploration.

The significance of this study lies in two key aspects. First, it identifies AC as a candidate natural compound with PI3K/AKT pathway modulatory activity, providing a basis for further development. In the context of ongoing efforts to discover effective and safe anti-gastric cancer agents from natural sources (Liu et al., 2025), AC represents a novel phytochemical scaffold worthy of investigation. Second, it adopts a “component-target-pathway” approach to offer a potential modern pharmacological basis for the traditional use of *A. decumbens*. Based on molecular dynamics simulations, we propose that AC exerts its effects through the modulation of the PI3Kα/AKT signaling axis, although this hypothesis requires experimental validation through the future studies outlined above. If confirmed, the unique structural characteristics of AC could provide a valuable scaffold for developing novel therapeutics, potentially contributing to the expanding arsenal of natural product-derived pathway inhibitors aimed at improving gastric cancer treatment outcomes.

## 5 Conclusion

This study demonstrates that AC, a bioactive compound derived from *A. decumbens*, exerts potent antitumor effects on human gastric cancer AGS cells by dual regulation of apoptosis and migration through PI3K/AKT pathway inhibition. Key experimental evidence revealed that AC concentration-dependently suppressed AGS cell proliferation, with an IC_50_ of 33.87 μM at 24 h as quantified by CCK-8 assay, indicating significant cytotoxicity. Mechanistically, AC triggered pro-apoptotic effects, elevating the apoptotic rate to 30.39% ± 1.45% (vs. 2.23% ± 0.13% in controls; ***P < 0.001) via Annexin V/PI staining, while concurrently inhibiting metastatic potential by reducing scratch healing rates to 19.07% ± 8.59% (vs. 38.56% ± 6.85% in controls; *P < 0.05). Long-term clonogenicity assays further confirmed its anti-proliferative efficacy, showing marked reduction in colony formation (**P < 0.01). Crucially, Western blot analysis established that these phenotypic alterations were mediated through dose-dependent suppression of the PI3K/AKT pathway: protein expression of PI3Kα decreased to 4.89% ± 1.59% (vs. 11.71% ± 1.40% in controls), total AKT1 to 6.23% ± 2.67% (vs. 13.90% ± 2.15%), and phosphorylated AKT1 (p-AKT1 at Ser473) to 5.75% ± 2.71% (vs. 29.98% ± 5.50%), with the p-AKT1/AKT1 activation ratio plummeting from 171.3% to 90.18% (**P < 0.01). These results mechanistically link AC’s antitumor actions to dual blockade of PI3Kα expression and AKT phosphorylation, disrupting downstream survival/migration signals. Collectively, our work suggests the potential of AC as a natural product that may target the PI3K/AKT axis. It achieves two primary contributions. First, it attempts to bridge traditional knowledge and modern molecular oncology by proposing a plausible “constituent-target-pathway” framework, thereby offering a preliminary molecular basis for further investigation into the traditional use of *A. decumbens*. Second, the integration of phenotypic assays with computational simulations exemplifies a multi-disciplinary strategy for researching natural products. These findings highlight the prospective value of phytochemicals in targeted therapy discovery and support the need for further studies to explore the translational potential of AC, including *in vivo* validation and an assessment of its potential synergistic effects within the complete herbal matrix.

## Data Availability

The datasets presented in this study can be found in online repositories. The names of the repository/repositories and accession number(s) can be found in the article/Supplementary Material.
