# Supplementary material for: Elucidating the dual regulation of apoptosis and migration by Ajugasterone C in AGS cells via the PI3K/AKT pathway
Source: Front Pharmacol. 2026 Jul 2;17:1823496. doi: 10.3389/fphar.2026.1823496 (PMC13373536; doi:10.3389/fphar.2026.1823496)
Supplement: Supplementary file 1 [file DataSheet1.docx]

Supplementary Figure 1: Inhibitory effect of AC on the proliferation of AGS and HGC-27 cells. (A) Dose-response curve showing the inhibitory effect of AC on AGS cell viability after 48 h of treatment. (B) Dose-response curve of AC on AGS cell viability after 72 h of treatment. (C) Dose-response curve of AC on HGC-27 cell viability after 24 h of treatment. The X-axis represents the logarithm of the Ajugasterone C concentration (Log [Ajugasterone C, µM]), and the Y-axis indicates the percentage of cell viability relative to the untreated control. The data points (dots) represent the mean values with 5 biological replicates per group (n=5), and the solid curve is the fitted dose-response curve. The region between two black dashed lines indicates the 95% confidence interval of the fit. The grey dashed line marks the 50% viability level. The intersection of this dashed line with the fitted curve determines the IC_50_.


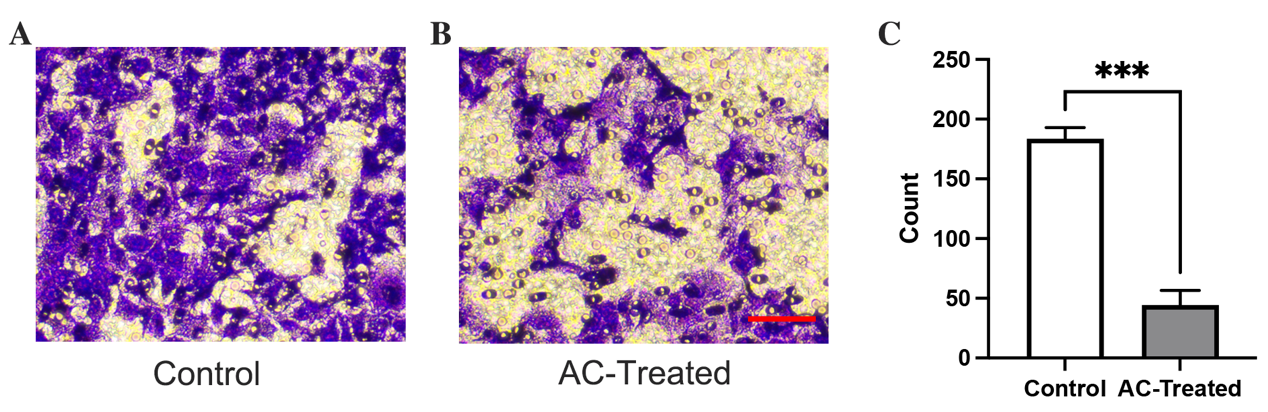


Supplementary Figure 2: Inhibition of cell proliferation in AC-treated groups. (A) Representative images of colony formation in the Control group. (B) Representative images of colony formation in the AC-Treated group. Cells were stained with 0.1% crystal violet. The red scale bar indicates 50 μm. (C) Quantitative analysis of the colony formation count. Data are presented as the mean ± SD from three independent experiments. Statistical significance was determined by Student's t-test. ****P* < 0.001 indicates significant differences compared with the Control group.


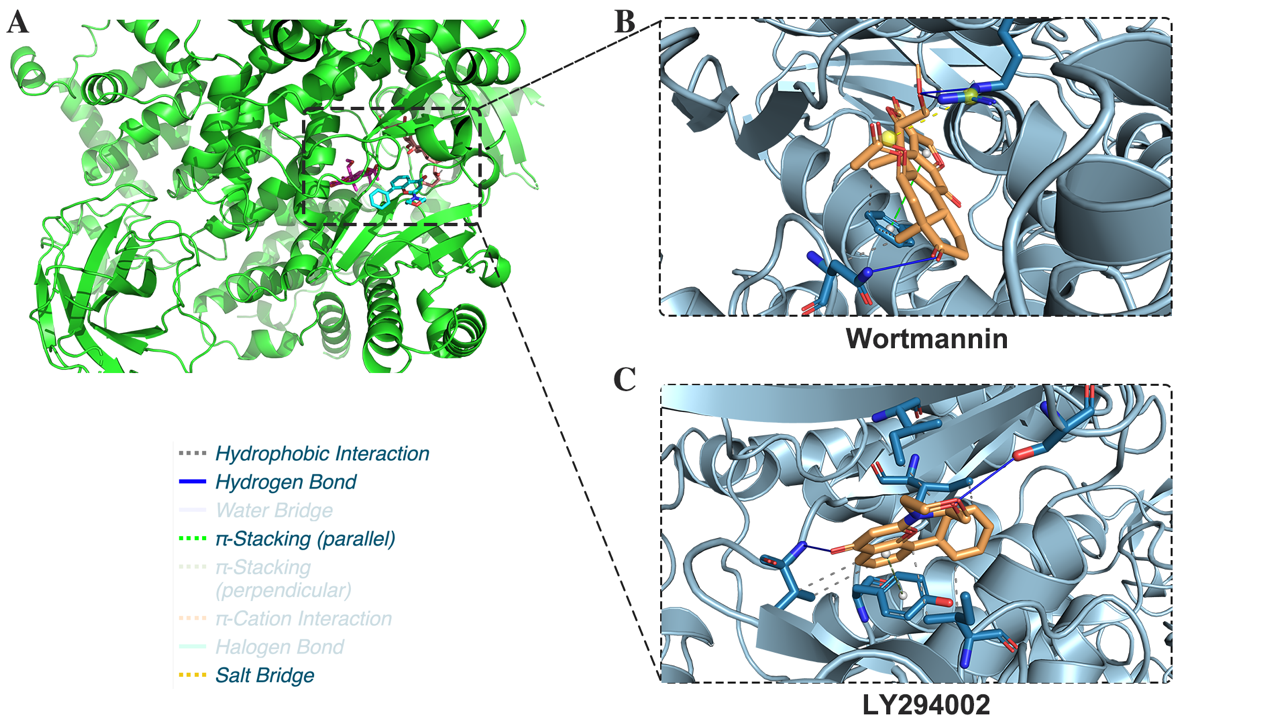


Supplementary Figure3: Molecular docking analysis of AC and its comparison with Wortmannin and LY294002. (A) Overall molecular docking schematic. The green ribbon represents the protein structure. The binding poses of Wortmannin, LY294002, and AC within the protein's active pocket are shown in light blue, magenta, and orange, respectively. (B) Local magnification of the binding pocket for Wortmannin. (C) Local magnification of the binding pocket for LY294002. The legend indicates various interaction forces: Hydrophobic Interaction (purple dots), Hydrogen Bond (blue solid line), Water Bridge (grey dashed line), π-Stacking (parallel and perpendicular, green and orange dotted lines), π-Cation Interaction (red dashed line), Halogen Bond (cyan solid line), and Salt Bridge (orange dashed line).

Supplementary Figure4: Molecular dynamics simulation results for the inhibitor LY294002. (A) Time evolution of the number of hydrogen bonds formed between the ligand and the protein throughout the simulation trajectory. (B) Distribution profile of donor-acceptor distances for the hydrogen bonds observed during the simulation. (C) Distribution profile of the angles (D-H···A) for the hydrogen bonds observed during the simulation. (D) RMSD of the protein backbone atoms relative to the initial structure, plotted as a function of simulation time, reflecting the overall structural stability of the complex. (E) Time evolution of the Rg of the complex, indicating changes in overall compactness and shape over the simulation period. (F) RMSF per residue for the protein backbone, highlighting regions of high flexibility (peaks) and low flexibility (troughs) during the simulation.

Supplementary Figure5: Molecular dynamics simulation results for the inhibitor Wortmannin. (A) Time evolution of the number of hydrogen bonds formed between the ligand and the protein throughout the simulation trajectory. (B) Distribution profile of donor-acceptor distances for the hydrogen bonds observed during the simulation. (C) Distribution profile of the angles (D-H···A) for the hydrogen bonds observed during the simulation. (D) RMSD of the protein backbone atoms relative to the initial structure, plotted as a function of simulation time, reflecting the overall structural stability of the complex. (E) Time evolution of the Rg of the complex, indicating changes in overall compactness and shape over the simulation period. (F) RMSF per residue for the protein backbone, highlighting regions of high flexibility (peaks) and low flexibility (troughs) during the simulation.

Supplementary Table 1. Hydrogen Bonds between the ligand (LY294002) and receptor

| Index | Residue | Amino | Distance H-A | Distance D-A | Donor Angle | Donor Atom | Acceptor Atom |
| --- | --- | --- | --- | --- | --- | --- | --- |
| 1 | 774A | SER | 3.10 | 3.71 | 111.66 | 6308[O3] | 8956[O3] |
| 2 | 851A | VAL | 3.06 | 3.93 | 130.11 | 7066[Nam] | 8955[O2] |

Supplementary Table 2. Hydrophobic Interactions between the ligand (LY294002) and receptor

| Index | Residue | Amino | Distance | Ligand Atom | Protein Atom |
| --- | --- | --- | --- | --- | --- |
| 1 | 800A | ILE | 3.70 | 8953 | 6578 |
| 2 | 848A | ILE | 3.63 | 8952 | 7046 |
| 3 | 848A | ILE | 3.53 | 8942 | 7045 |
| 4 | 851A | VAL | 3.92 | 8943 | 7072 |
| 5 | 851A | VAL | 3.82 | 8941 | 7070 |
| 6 | 932A | ILE | 3.36 | 8949 | 7796 |
| 7 | 932A | ILE | 3.86 | 8939 | 7798 |

Supplementary Table 3. π-Stacking between the ligand (LY294002) and receptor

| Index | Residue | Amino | Distance | Angle | Offset | Stacking Type | Ligand Atom |
| --- | --- | --- | --- | --- | --- | --- | --- |
| 1 | 836A | TYR | 4.85 | 72.94 | 0.42 | T | 8936, 8937, 8939, 8941, 8942, 8943 |

Supplementary Table 4. Hydrogen Bonds between the ligand (Wortmannin) and receptor

| Index | Residue | Amino | Distance H-A | Distance D-A | Donor Angle | Donor Atom | Acceptor Atom |
| --- | --- | --- | --- | --- | --- | --- | --- |
| 1 | 756A | ASN | 2.33 | 3.26 | 132.87 | 6130[Nam] | 8959[O2] |
| 2 | 818A | ARG | 1.91 | 3.05 | 158.43 | 6759[Ng+] | 8964[O3] |
| 3 | 818A | ARG | 2.37 | 3.25 | 128.44 | 6762[Ng+] | 8964[O3] |

Supplementary Table 5. Hydrophobic Interactions between the ligand (Wortmannin) and receptor

| Index | Residue | Amino | Distance | Ligand Atom | Protein Atom |
| --- | --- | --- | --- | --- | --- |
| 1 | 666A | PHE | 3.74 | 8949 | 5200 |
| 2 | 666A | PHE | 3.72 | 8944 | 5202 |
| 3 | 756A | ASN | 3.60 | 8944 | 6128 |

Supplementary Table 6. π-Stacking between the ligand (Wortmannin) and receptor

| Index | Residue | Amino | Distance | Angle | Offset | Stacking Type | Ligand Atom |
| --- | --- | --- | --- | --- | --- | --- | --- |
| 1 | 666A | PHE | 4.67 | 24.34 | 0.98 | P | 8945, 8948, 8950, 8952, 8958 |

Supplementary Table 7. Salt Bridges between the ligand and receptor

| Index | Residue | Amino | Distance | Ligand Group | Ligand Atoms |
| --- | --- | --- | --- | --- | --- |
| 1 | 818A | ARG | 4.32 | Carboxylate | 8962, 8963 |
